# Understanding the effect of indoor air pollution on pneumonia in children under 5 in low- and middle-income countries: a systematic review of evidence

**DOI:** 10.1007/s11356-018-3769-1

**Published:** 2018-12-19

**Authors:** Enemona Emmanuel Adaji, Winifred Ekezie, Michael Clifford, Revati Phalkey

**Affiliations:** 1 Division of Epidemiology and Public Health, University of Nottingham, Nottingham City Hospital, Clinical Sciences Building, Hucknall Road, Nottingham, NG5 1PB UK; 2 Faculty of Engineering, University of Nottingham, Nottingham, UK; 3 Climate Change and Human Health Group, Institute for Public Health, University of Heidelberg, Heidelberg, Germany

**Keywords:** Indoor air pollution, Black carbon, Particulate matter, Carbon monoxide, Pneumonia, Children under 5, Low- and middle-income countries

## Abstract

Exposure to indoor air pollution increases the risk of pneumonia in children, accounting for about a million deaths globally. This study investigates the individual effect of solid fuel, carbon monoxide (CO), black carbon (BC) and particulate matter (PM)_2.5_ on pneumonia in children under 5 in low- and middle-income countries. A systematic review was conducted to identify peer-reviewed and grey full-text documents without restrictions to study design, language or year of publication using nine databases (Embase, PubMed, EBSCO/CINAHL, Scopus, Web of Knowledge, WHO Library Database (WHOLIS), Integrated Regional Information Networks (IRIN), the World Meteorological Organization (WMO)-WHO and Intergovernmental Panel on Climate Change (IPCC). Exposure to solid fuel use showed a significant association to childhood pneumonia. Exposure to CO showed no association to childhood pneumonia. PM_2.5_ did not show any association when physically measured, whilst eight studies that used solid fuel as a proxy for PM_2.5_ all reported significant associations. This review highlights the need to standardise measurement of exposure and outcome variables when investigating the effect of air pollution on pneumonia in children under 5. Future studies should account for BC, PM_1_ and the interaction between indoor and outdoor pollution and its cumulative impact on childhood pneumonia.

## Introduction

Pneumonia is an inflammatory disease affecting the lung. It is characterised by an accumulation of fluid in the alveolus, resulting in the obstruction of normal breathing (CDC 2016; Walker et al. 2013). Pneumonia is caused predominantly but not solely by bacteria, viruses and fungi (CDC 2016; Walker et al. 2013). In children, bacterial pneumonia is mostly caused by *Streptococcus pneumoniae* whilst *Haemophilus influenzae* type b (Hib) is the next largest cause (CDC 2016; Walker et al. 2013). Respiratory syncytial virus mainly causes viral pneumonia whilst *Pneumocystis jirovecii* is the major cause of fungal pneumonia in children (Liu et al. 2015). Globally, pneumonia is the major cause of paediatric mortality, especially in children under 5 (McCollum et al. 2015; UNICEF 2016; Walker et al. 2013). A 2015 report by UNICEF has shown that one in six children died from pneumonia before the age of 10 particularly in the poorest parts of low- and middle-income countries (UNICEF 2016). Sub-Saharan Africa and South Asia are the regions with the highest number of pneumonia deaths. The number of deaths in these regions has continued to rise, increasing from 77% in 2000 to 82% in 2015. This staggering number of worldwide pneumonia deaths is concentrated mostly in four countries since 2000: the Democratic Republic of Congo, India, Nigeria and Pakistan (UNICEF 2016).

The high deaths from pneumonia can also be linked to poverty. Looking closely, these deaths are concentrated amongst the poorest populations. Sixty-two per cent of the world’s under-5 population can be found in low- and middle-income countries. However, 90% of the global deaths from pneumonia still originate from these regions.

The very poorest countries carry a disproportionate share of the burden of death: more than 30% of all pneumonia and diarrhoea deaths are concentrated in low-income countries, yet these countries are home to only 15% of the world’s under-5 population (UNICEF 2016). These regions are also characterised by reduced access to quality health care, nutrition and basic environmental hygiene which contributes to exacerbating the disease incidence (UNICEF 2016). Pneumonia is preventable and so is the related morbidity (Chopra et al. 2013; Qazi et al. 2015).

There were over 4.1 million deaths and 106 million daily-adjusted life years (DALYs) lost worldwide due to long-time exposure to particulate matter (PM)_2.5_ in 2016. Homes that depend on the daily use of solid fuels been exposed to PM_2.5_ levels six times higher than the least severe WHO Interim Air Quality Target and as high as 20 times higher than the recommended WHO Air Quality Guideline of 10 μg/m^3^ for PM_2.5_ (Health Effects Institute 2018). The risk of developing pneumonia in children is doubled following exposure to air pollution, thereby accounting for over 920,000 deaths globally, caused by pneumonia (UNICEF 2016). Pollutants compromise the host’s immune response against invading pathogens in the respiratory tract. The epithelial cells lining the alveolar are specialised in secreting cytokines and radicals in response to foreign evading bodies (Hussey et al. 2017; Smith et al. 2000). These mediate the recruitment of inflammatory cells such as macrophages and phagocytes to the site of evasion, which then engulf and digest these foreign organisms. However, high levels of air pollution can lead to a compromise in this sterilisation and filtration mechanism of the respiratory tract, therefore increasing the risk of the development of acute lower respiratory infections (ALRIs) (Smith et al. 2000).

Furthermore, the mucociliary apparatus and cellular immune defences have also been shown to be significantly reduced by nitrogen dioxide (Carvalho-Oliveira et al. 2015; Caswell 2013; Smith et al. 2000). Most characterised types of pollutants belong to a broad class called “particulate matter” (Smith et al. 2000). PM has been previously reported as the key mediator of inflammation and compromised immune defences that are linked to the pathogenesis of ALRIs (Bauer et al. 2012; Lee et al. 2015; Smith et al. 2000). More recently, black carbon (BC), which is a component of PM, has been reported to be the main mediator of the effects of pollutants (Smith et al. 2000). BC has been reported to significantly affect the behaviour of *S. pneumoniae* by altering the structure and proteolytic degradation of the biofilm, therefore promoting its tolerance to multiple antibiotics. Finally, BC promotes the spread of bacteria to the lungs and consequently exacerbates the disease occurrence (Baumgartner et al. 2014; Hussey et al. 2017). Although these studies have looked at the pathological relationship between the PM, BC and respiratory diseases, the consensus on disease incidence remains contested.

Indoor pollution is not restricted to habitats of rural settlements and poor people (DD 2013; Hulin et al. 2010; Jung et al. 2012; Ram et al. 2014; Sharma et al. 1998; Shibata et al. 2014). Studies have shown the association between respiratory illness and indoor activities and features such as new painting and wall covers (Emenius et al. 2004; Garrett et al. 1998; Jaakkola et al. 2004), gas appliances (Agabiti et al. 1999; Belanger et al. 2006; Pilotto et al. 1999) or exposure to particles from tobacco smoke or heating system utilising coal (Gonzalez-Barcala et al. 2013; Nandasena et al. 2013).

Children are at higher risk because of their increased resting metabolism and a higher degree of aerobic metabolism relative to their size compared to adults (American Thoracic Society 2004; Chance 2001). Children are continuously undergoing organ development; this coupled with an elevated surface area per unit body weight increases the oxygen demand and respiratory rates (Moya et al. 2004). Furthermore, children have narrower airways compared to adults; therefore, whilst a pollutant may cause a mild irritation in the adult airways, in a child, it can potentially be a more substantial obstruction (Bruce et al. 2013).

Most of the research studies carried out in low- and middle-income countries mainly depend on questionnaires for data collection. These studies are limited because they often depend on self-reported questionnaires to understand exposures related to indoor air pollution and health outcomes, which are subject to several inherent biases (Broor et al. 2001; Sharma et al. 1998; Dhimal et al. 2010; Kelly et al. 2015; Mahalanabis et al. 2002; Karki et al. 2014; PrayGod et al. 2016; Mustapha et al. 2011. In recent years, the emergence of specific instrument allowing the measurement and quantification of indoor air quality has enabled researchers to identify pollutants involved in causing diseases in rural and urban areas in France (Hulin et al. 2010) and low- and middle-income countries such as Eastern Indonesia (Shibata et al. 2014).

We found eight reviews that investigated the impact of indoor air pollution on childhood pneumonia in children under 5. However, most of them investigated indoor air pollution as a single outcome (Bruce et al. 2013; Buchner and Rehfuess 2015; Dherani et al. 2008; Jackson et al. 2013; Rudan et al. 2008; Smith et al. 2000; Sonego et al. 2015; Zar and Ferkol 2014). However, they do not investigate the granularity of exposures. Therefore, this systematic review aims to segregate indoor air pollution into its different constituents and investigate the evidence available on how solid fuel use, PM_2.5_, carbon monoxide (CO), BC and other risk factors individually affect pneumonia in children under 5 in low- and middle-income countries.

## Methods

Five electronic databases for published literature were used to identify peer-reviewed articles. These include Embase, PubMed, EBSCO/CINAHL, Scopus and Web of Knowledge. Additionally, four databases were used to identify grey literature, which included WHO Library Database (WHOLIS), Integrated Regional Information Networks (IRIN), the World Meteorological Organization (WMO)-WHO and Intergovernmental Panel on Climate Change (IPCC). Medical Subject Headings (MeSH), free-text terms and keywords used in the search include pollution (pollution, air pollution, indoor air pollution, carbon monoxide, carbon dioxide, nitrogen monoxide, nitrogen dioxide, particulate matter, sulphur dioxide, ozone, volatile organic compound and black carbon). These were combined with Boolean operators and/or with health (pneumonia, acute lower respiratory infection, respiratory health). The search strategy was customised to each database (Appendix 1). A reference list of selected papers was hand searched for relevant studies. The inclusion criteria were set at full-text articles investigating the effect of indoor air pollution on pneumonia in children under 5 residing in low- and middle-income countries (as defined by the World Bank (The World Bank 2017)), with a sample size above 100. The inclusion was not restricted by study design, language or year of publication.

**Fig. 1:**
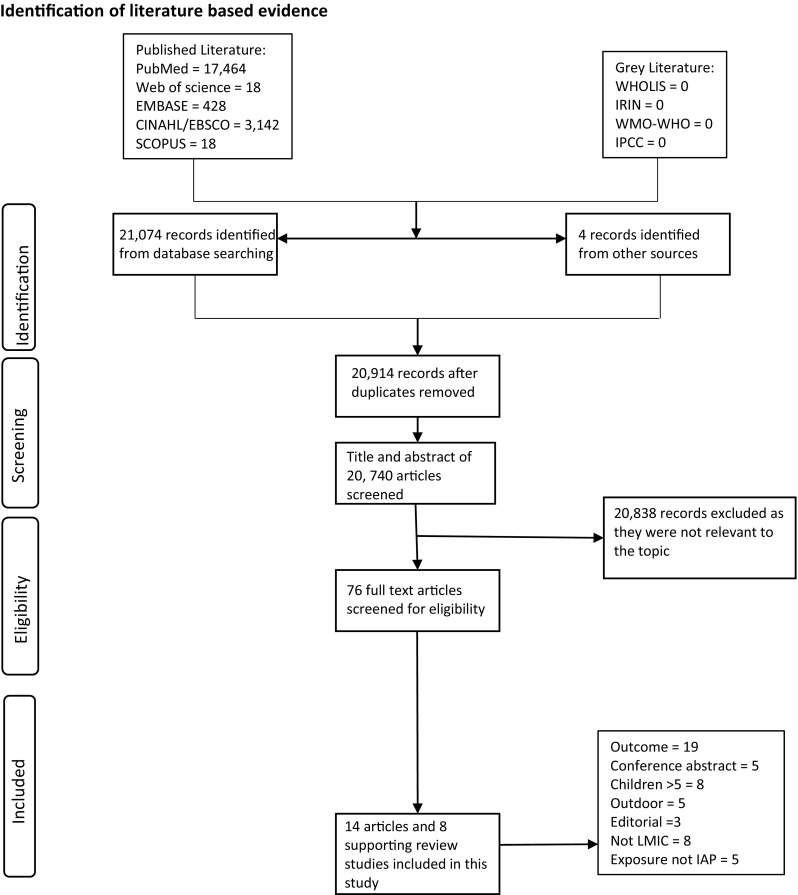
The flow of studies from identification to data extraction from databases based on the PRISMA guidelines

The electronic search resulted in 21,074 papers. All papers were imported into Endnote, and 164 duplicates were removed automatically. After title and abstract screening by two independent reviewers (EA and WE) 20,838 papers were excluded due to lack of relevance to the current study. Seventy-six articles were selected for full-text review, of which 64 were rejected (17 not in the appropriate age range, 12 not focused on indoor air pollution, 15 measuring an irrelevant outcome, 12 non-full text articles and eight conducted in high-income countries). Eight review papers were considered relevant and included as supporting documents in the final review (Fig. 1). Disagreements throughout the review process were resolved with mutual consent.

### Quality assessment and risk of bias

The PRISMA guidelines were used for the review process (Liberati et al. 2009). The Newcastle-Ottawa Scale (Stang 2010) was used to assess the methodological quality and the risk of bias in individual studies (see Appendixes 5 and 6). The risk of bias for each study was divided into low, medium and high across the following domains: selection of participants (selection bias), sample size justification (selection bias), outcome measurement and confounding adjustment.

## Results

The 22 studies included in this review included *nine case-control studies* (Bassani et al. 2010; Broor et al. 2001; Dionisio et al. 2012; Howie et al. 2016; Karki et al. 2014; Mahalanabis et al. 2002; PrayGod et al. 2016; Ram et al. 2014; Sharma et al. 1998); *one cohort study* (Kelly et al. 2015); one cross-sectional study (Dhimal et al. 2010); *one mixed-method study, using both cross-sectional and case-control methods* (Shibata et al. 2014); *two randomised trials* (Mortimer et al. 2017; Smith et al. 2011); and *eight review papers* (Bruce et al. 2013; Buchner and Rehfuess 2015; Dherani et al. 2008; Jackson et al. 2013; Rudan et al. 2008; Smith et al. 2000; Sonego et al. 2015; Zar and Ferkol 2014).

The country with the highest number of studies was India with four studies (Bassani et al. 2010; Broor et al. 2001; Mahalanabis et al. 2002; Sharma et al. 1998), followed by Nepal with two studies (Dhimal et al. 2010; Karki et al. 2014), the Gambia with two studies (Dionisio et al. 2012; Howie et al. 2016) and one study each from Bangladesh (Ram et al. 2014), Tanzania (PrayGod et al. 2016), Indonesia (Shibata et al. 2014), Malawi (Mortimer et al. 2017), Guatemala (Smith et al. 2011) and Botswana (Kelly et al. 2015). The studies were conducted between 1994 and 2017 with the majority covering the period 2007–2017.

The sample size and age of children varied in each study with a median size of 522 participants and from age 0 to 60 months, respectively. Nine (64%) studies were carried out in peri-urban cities, with only two studies looking at both urban and rural (Bassani et al. 2010) and peri-urban and rural (Mortimer et al. 2017; Smith et al. 2011; Karki et al. 2014). Thirteen studies collected primary data (Mortimer et al. 2017; Smith et al. 2011; Broor et al. 2001; Dionisio et al. 2012; Howie et al. 2016; Karki et al. 2014; Kelly et al. 2015; Mahalanabis et al. 2002; PrayGod et al. 2016; Ram et al. 2014; Sharma et al. 1998; Shibata et al. 2014), with only two using secondary data (Bassani et al. 2010; Dhimal et al. 2010).

Table 1 provides an overview of the included studies and their main findings. Solid fuel use was found to be significantly associated with childhood pneumonia in a majority of the studies (eight studies, 57%) (Bassani et al. 2010; Broor et al. 2001; Dhimal et al. 2010; Karki et al. 2014; Kelly et al. 2015; Mahalanabis et al. 2002; PrayGod et al. 2016; Sharma et al. 1998). Neither of the two studies investigating exposure to CO reported a significant association with pneumonia as an outcome (Dionisio et al. 2012; Howie et al. 2016). Similarly, exposure to PM_2.5_ showed no significant association in two studies (17%) that actually measured PM_2.5_ against using a proxy indicator (Ram et al. 2014; Shibata et al. 2014). Two trials also showed that interventions providing improved cookstoves did not reduce the risk of pneumonia in children (Mortimer et al. 2017; Smith et al. 2011).

**Table 1:** Studies showing the association between indoor air pollution and childhood pneumonia

No.	Country	Name/year of study	Setting	Study design	Study period	Primary/secondary data	Age in months	Exposure	Data collected using	Sample size	Outcome parameter	Main result
1	India	Broor et al. (2001)	Peri-urban	Case-control	March 1995–February 1997	Primary	< 60	Solid fuel use	Questionnaire	512	Single disease episode	Cooking with any other type of fuel other than LPG (OR 2.5, 1.51–4.16)
2	India	Sharma et al. (1998)	Peri-urban	Case-control	November 1994–February 1995	Primary	< 12	Fuel used for cooking	Questionnaire	642	Multiple disease episode	Pneumonia was the most common illness in both fuel groups used at home for both wood and kerosene
3	India	Bassani et al. (2010)	Urban/rural	Case-control	February 1998	Secondary	< 48	Solid fuel use	Survey data	616,391	Mortality and single disease episode	Generally, children (0–4 years) with pneumonia had a higher reported solid fuel use compared to children without pneumonia (boys: PR 1.5, 1–2.4; girls: PR 1.9, 1.1–3.3)
4	Nepal	Dhimal et al. (2010)	Peri-urban	Cross-sectional	October 2008–January 2009	Primary and secondary	< 60	Fuel used for cooking	Questionnaire	545,777	Single disease episode	The solid biomass fuel was the primary source of energy for cooking which attributed to about 50% of the burden of pneumonia in children
5	Botswana	Kelly et al. (2015)	Urban/peri-urban	Cohort study	April 2012–April 2014	Primary	< 24	Wood smoke exposure	Questionnaire	284	Single disease episode and mortality	The risk of failure to respond to treatment at 48 h was increased in households that used wood as a cooking fuel (RR 1.44, 95% CI 1.09–1.92, *P* = 0.01). This effect was observed in undernourished children (*P* = 0.02)
6	Gambia	Dionisio et al. (2012)	Urban/peri-urban	Case-control	July 2007–January 2011	Primary	< 60	Exposure to CO	CO measurement questionnaire	1181	Single disease episode	There was an increased risk of pneumonia (OR 4.2, 3.1–5.7) in the rainy season compared to the dry season. Households where firewood or charcoal was purchased exposed children 2.0 (1.2–3.2) or 3.8 (2.1–7.1) times more to indoor air pollution compared to households that collected firewood
7	Gambia	Howie et al. (2016)	Urban/peri-urban	Case-control	June 2007-September 2010	Primary	< 60	Exposure to CO	CO measurement questionnaire	1581	Single disease episode	No association was found between CO exposure and childhood pneumonia. However, bed sharing with someone with a cough and severe pneumonia (OR 5.1, 3.2–8.2) and non-severe pneumonia (OR 7.3, 4.1–13.1). Undernutrition was associated with childhood pneumonia (OR 8.7, 4.2–17.8)
8	Indonesia	Shibata et al. (2014)	Urban	Cross-sectional/case-control	June 2011–June 2012	Primary	< 60	Measured PM_2.5_ and PM_10_	PM_2.5_ measurement questionnaire	461	Single disease episode	Hourly sampling showed significant differences in PM_2.5_ and PM_10_ concentration between households in which children with pneumonia lived compared with controls
9	Bangladesh	Ram et al. (2014)	Urban	Case-control	March 2009–March 2010	Primary	< 60	Exposure to PM_2.5_	PM_2.5_ measurement questionnaire	994	Single disease episode	PM_2.5_ was not significantly associated with pneumonia. However, crowding, aluminium roofing in living space, households with lower socioeconomic status and being a boy were associated with pneumonia
10	India	Mahalanabis et al. (2002)	Urban/peri-urban	Case-control	December 1997–November 1998	Primary	< 35	Risk factors	Questionnaire	262	Single disease episode	Solid fuel use with OR 3.97, 2–7.88, compared to not using any solid fuel for cooking
11	Nepal	Karki et al. (2014)	Peri-urban and rural	Case-control	June 2012–May 2013	Primary	< 60	Risk factors	Questionnaire	200	Single disease episode	Using solid fuel with location within living area (OR 3.76, 1.20–11.82)
12	Tanzania	PrayGod et al. (2016)	Urban/peri-urban	Case-control	May 2013–March 2014	Primary	< 60	Behaviour	Questionnaire	117	Single disease episode	Cooking indoors increased the risk of developing severe pneumonia (OR 5.5, 1.4–22.1) compared to cooking outdoors
13	Malawi	Mortimer et al. (2017)	Rural	Randomised control trial	December 2013–February 2016	Primary	< 60	Biomass smoke	Questionnaire and exposure measurement	10,543	Mortality and single disease	Cleaner burning biomass-fuelled cookstoves did not reduce the risk of pneumonia in young children under 5 in Malawi
14	Guatemala	Smith et al. (2011)	Rural	Randomised control trial	October 2002–December 2004	Primary	< 18	Household air pollution	Questionnaire and exposure measurement	534	Mortality and single disease episode	Reduction of wood smoke exposure with chimney stoves did not significantly reduce pneumonia in children under 5

Pollution measurements within the studies were compared with the WHO-approved standard (WHO 2017). Measurements were taken over 24 h amongst studies, and repeated measurements were done between 2 and 3 months (Dionisio et al. 2012). Five studies that accounted for seasonal variation matched their study participants according to the season of recruitment (Bassani et al. 2010; Dionisio et al. 2012; Howie et al. 2016; Sharma et al. 1998; Shibata et al. 2014). Given the wide variation in the objectives and designs of the included studies, it was difficult to summarise the findings. We, therefore, describe the results in four subsections according to exposures investigated: solid fuel use, CO, PM_2.5_ and other general risk factors identified.

### Exposure to solid fuel use

Eight (57%) studies investigated the association between solid fuel use and childhood pneumonia. Six studies found positive associations between solid fuel use and pneumonia in children. Broor et al. (2001), from India, reported that any cooking fuel other than liquid petroleum gas was associated with pneumonia in children under 5 (odds ratio (OR) 2.5, 1.51–4.16). Karki et al. (2014), from Nepal, identified a significant association for the type of cooking fuel (chulo) used in the household with increased childhood pneumonia (OR 3.76, 1.20–11.82). Whilst two trials also showed that interventions providing improved cookstoves did not reduce the risk of pneumonia in children (Mortimer et al. 2017; Smith et al. 2011).

Furthermore, Bassani et al. (2010), from India, reported that solid fuel use significantly increased all-cause child mortality for boys (prevalence ratio (PR) 1.30, 1.08–1.56) and girls (PR 1.33, 1.12–1.58); also, association with non-fatal pneumonia was observed for boys (PR 1.54, 1.01–2.35) and girls (PR 1.94, 1.13–3.33). Kelly et al. (2015), from Botswana, found that houses using wood as fuel showed an increased risk of failure to respond to pneumonia treatment at 48 h (RR 1.44, 1.09–1.92). Dhimal et al. (2010), from Nepal, established that a total of 1284 DALYs were lost due to pneumonia and half of it was due to smoke from solid fuels used for cooking indoors. Sharma et al. (1998), from India, stated that pneumonia was common across all study groups using both kerosene and wood as sources of fuel. In contrast, PrayGod et al. (2016), from Tanzania, reported that fuel type was not significantly associated with pneumonia. Shibata et al. (2014), from Indonesia, also confirmed this by reporting that the use of unhealthy cooking fuels was not significantly associated with pneumonia.

### Exposure to CO

Two out of the 12 studies investigated the effect of CO on childhood pneumonia. Both studies from the Gambia, Howie et al. (2016) and Dionisio et al. (2012), reported no association between pneumonia and individual CO exposure as a measure of household air pollution.

### Exposure to PM_2.5_

Ten out of the 12 studies investigated the effect of particulate matter with particles less than or equal to 2.5 μm in diameter (PM_2.5_) on childhood pneumonia. Out of the ten studies that looked at PM_2.5_, only two directly measured PM_2.5_ (Ram et al. 2014; Shibata et al. 2014), whilst eight used solid fuel use as a proxy indicator for PM_2.5_ (Bassani et al. 2010; Broor et al. 2001; Dhimal et al. 2010; Karki et al. 2014; Kelly et al. 2015; Mahalanabis et al. 2002; PrayGod et al. 2016; Sharma et al. 1998). The two studies that directly measured PM_2.5_ did not find any association, whilst the eight studies that used solid fuel as a proxy all reported associations.

Ram et al. (2014), from Bangladesh, found no association between PM_2.5_ and childhood pneumonia. However, they found an increased level of exposure to particulate matter in the households of cases than in control households. Particularly looking at cooking spaces in case households, PM_2.5_ was found to be 1.64 times more likely to be greater than 100 μg/m^3^ for more than 4 h compared to control households. Similarly, PM_2.5_ was found to be 1.70 times more likely to be greater than 250 μg/m^3^ for more than 1 h in cases compared to control households. Furthermore, in living spaces, PM_2.5_ was found to be 1.65 times more likely to be greater than 250 μg/m^3^ for more than 30 min in cases compared to control households. Also, increasing the duration of PM_2.5_ exposure greater than 100 μg/m^3^ or 250 μg/m^3^ showed no association with increased pneumonia incidence. In multivariate analysis, no association was found in any of the PM_2.5_ measures and pneumonia. Shibata et al. (2014), from Indonesia, also confirmed this, as there was no association found between PM_2.5_ levels when cases were compared with controls.

### General risk factors associated with childhood pneumonia

Besides air quality measurements, all 12 studies described general risk factors (Table 2), which included socioeconomic factors, malnutrition, cooking location, bed sharing, the number of people sleeping in a room, the number of siblings, cross-ventilation and season. Howie et al. (2016) found an association between malnutrition and pneumonia (OR 8.7, 4.2–17.8). Karki et al. (2014), from Nepal, found that the risk of pneumonia was increased by up to four times in houses where cooking was done indoors (OR 3.76, 1.20–11.82).

**Table 2:** Overview of included studies and summary of variables adjusted for within each study

No.	Country	Study			Adjusted for																		
		Name/year of study	Definition of air pollution	Pneumonia measurement	Access to care	Immunisation	Malnutrition	Exclusive breastfeeding	LBW	No. of siblings	Marital status	Occupation/SES	Parents smoke	Parent’s education	Religion	Kitchen location	Cooking fuel type	Cook area	No sleeps in room	Ventilation	House material	Source of drinking water	Season
1	India	Broor et al. (2001)	Use of solid fuel	Syndromic	✔	✔^†^	✔^†^	✔^†^		✔		✔	✔	✔			✔^†^				✔		
2	Botswana	Kelly et al. (2015)	Use of wood as fuel	Syndromic	✔		✔^†^		✔			✔		✔			✔^†^		✔			✔	
3	India	Sharma et al. (1998)	Types of fuel used at home	Syndromic	✔	✔	✔^†^					✔		✔^†^			✔^†^		✔				✔
4	Nepal	Karki et al. (2014)	Condition of home environment	Presumptive	✔	✔		✔			✔	✔	✔			✔^†^	✔	✔^†^					
5	India	Mahalanabis et al. (2002)	Condition of home environment	Presumptive	✔			✔	✔	✔		✔^†^	✔	✔			✔^†^				✔		
6	Bangladesh	Ram et al. (2014)	Concentration of PM_2.5_	Presumptive	✔							✔^†^	✔					✔	✔^†^	✔^†^	✔^†^		
7	India	Bassani et al. (2010)	Use of solid fuel							✔^†^		✔		✔^†^	✔		✔^†^	✔			✔^†^	✔	✔
8	Gambia	Dionisio et al. (2012)	Exposure to CO	Presumptive	✔							✔^†^	✔				✔^†^	✔					✔^†^
9	Gambia	Howie et al. (2016)	Exposure to CO	Presumptive	✔	✔	✔^†^	✔	✔			✔		✔			✔		✔			✔	✔
10	Tanzania	PrayGod et al. (2016)	Condition of home environment	Both syndromic and presumptive	✔	✔	✔				✔	✔	✔	✔	✔	✔	✔	✔^†^	✔				
11	Nepal	Dhimal et al. (2010)	Types of fuel used at home	Both syndromic and presumptive	✔			✔				✔	✔				✔						
12	Indonesia	Shibata et al. (2014)						✔^†^				✔	✔	✔			✔						✔
13	Malawi	Mortimer et al. (2017)	Types of fuel used at home	Both syndromic and presumptive	✔	✔	✔			✔		✔	✔			✔	✔	✔				✔	✔
14	Guatemala	Smith et al. (2011)	Exposure from type of fuel used at home	Both syndromic and presumptive	✔			✔				✔	✔	✔		✔	✔	✔	✔				
				Total	(15, 79%)	(8, 42%)	(8, 42%)	(10, 53%)	(6, 32%)	(7, 37%)	(2, 11%)	(19, 100%)	(13, 68%)	(14, 74%)	(2, 11%)	(5, 26%)	(15, 79%)	(8, 42%)	(9, 47%)	(2, 11%)	(4, 21%)	(5, 26%)	(8, 42%)

^†^Associated variables

Mahalanabis et al. (2002), from India, described a strong association between bed sharing and severe pneumonia (OR 5.1, 3.2–8.2) and non-severe pneumonia (OR 7.3, 4.1–13.1). Howie et al. (2016), from the Gambia, found an association between severe pneumonia (OR 5.1, 3.2–8.2) and non-severe pneumonia (OR 7.3, 4.1–13.1) and bed sharing with someone with a cough.

Ram et al. (2014), from Bangladesh, reported sleeping in a room with four or more people was significantly associated with pneumonia (OR 1.60, 1.18–2.18). However, PrayGod et al. (2016), from Tanzania, reported no significant association between the number of people sleeping in a room and pneumonia (OR 1.1, 0.7–1.7).

Mahalanabis et al. (2002), from India, did not find any association between numbers of siblings and the increase in disease episodes. However, Broor et al. (2001) and Bassani et al. (2010), both from India, reported having four or more siblings was significantly associated with all-cause mortality for both boys (PR 1.44, 1.18–1.76) and girls (PR 1.76, 1.43–2.10).

Ram et al. (2014), from Bangladesh, reported that having a cross-ventilated living area would be protective (OR 0.72, 0.53–0.98). Cross-ventilation in the living spaces of case households was 28% less than that in control households.

Five studies investigated seasonality. Bassani et al. (2010), from India, reported solid fuel use to be higher in children who died compared to living children between ages 1 and 4 in the same geographical area in winter months. Dionisio et al. (2012), from the Gambia, also reported that cooking with biomass fuel indoors or in an enclosed cookhouse rose to 84% during the rainy season compared to the dry season. However, Howie et al. (2016), from the Gambia, only matched participants based on seasons to account for the influence it might have on the study. Finally, Sharma et al. (1998), from India, and Shibata et al. (2014), from Indonesia, only took measurements during the winter and dry seasons, respectively, accepting that the complete seasonal influence within these studies is unknown.

### Supporting studies on indoor air pollution and pneumonia

Eight review papers (Bruce et al. 2013; Buchner and Rehfuess 2015; Dherani et al. 2008; Jackson et al. 2013; Rudan et al. 2008; Smith et al. 2000; Sonego et al. 2015; Zar and Ferkol 2014) included six systematic reviews and two meta-analyses. All eight studies tried to understand factors responsible for childhood respiratory infections within low- and middle-income countries. All eight studies (Table 3) reported indoor pollution as an important risk factor for the increase in childhood pneumonia. Sonego et al. (2015) reported OR of 3.02 (2.11–4.31). The study of Zar and Rudans also concluded that improving nutrition, comprehensive immunisation, reduction in household crowding, avoidance of smoking, reduction in exposure to indoor pollutants and tackling of HIV/AIDS incidence in low- and middle-income countries can help in the prevention of pneumonia in children.

**Table 3:** Reviews focused on the association between indoor air pollution and childhood pneumonia

No.	Author (year)	Study design	Country	Number of studies	Exposure	Outcome	Key findings
1	Jackson et al. (2013)	Systematic review	Developing countries	36	Use of biomass fuel for cooking	Pneumonia	Exposure to indoor air pollution (OR 1.57, 1.06–2.31)
2	Dherani et al. (2008)	Meta-analysis and systematic review	Developing countries	25	Behaviour and environment (fuel use)	Pneumonia (ALRI)	Indoor air pollution is associated with pneumonia (OR 1.8, 1.5–2.2)
3	Smith et al. (2000)	Critical review	Papua New Guinea, Kenya, India, Nepal, China, Gambia	18	Indoor air pollution	Pneumonia (ALRI)	Confirms an association between indoor air pollution and childhood pneumonia, particularly in households using biomass fuels
4	Sonego et al. (2015)	Systematic review	LMIC as defined by the World Bank	77	Risk factors	Pneumonia (ALRI)	Confirms an association between indoor air pollution and childhood pneumonia (OR 3.02, 2.11–4.31)
5	Zar and Ferkol (2014)	Review	LMIC	–	Environmental risk factor, including indoor air pollution	Pneumonia	Improving nutrition, comprehensive immunisation, reduction in household crowding, avoidance of smoking, reduction in exposure to indoor pollutants and tackling of HIV/AIDS incidence in low- and middle-income countries can help in the prevention of pneumonia in children
6	Bruce et al. (2013)	Systematic review and meta-analysis	Global reports on developing countries	26	Solid fuel used for cooking	Pneumonia (ALRI)	Eliminating exposures to indoor air pollution might considerably reduce the risk of pneumonia complications, including fatality
7	Rudan et al. (2008)	Systematic review	Global reports on developing countries	28	Indoor air pollution	Pneumonia (ALRI)	Show an association between indoor air pollution and childhood pneumonia (OR 1.8)
8	Buchner and Rehfuess (2015)	A cross-sectional multi-country analysis	Benin, Burkina Faso, Cameroon, Ethiopia, Ghana, Guinea, Kenya, Madagascar, Mali, Malawi, Mozambique, Namibia, Niger, Senegal, Tanzania, Uganda, Zambia and Zimbabwe	–	Risk factors (indoor air pollution)	Pneumonia (ALRI)	Show an association between indoor air pollution and childhood pneumonia (OR 2.17, 1.09–4.33)

Buchner and Rehfuess (2015) found that indoor cooking and season were significantly associated with childhood pneumonia with OR during the rainy season to be 1.80 (1.30–2.50) and OR during the dry season to be 1.51 (1.09–2.10). Bruce et al. (2013) focused on the type of fuel used and exposure to biomass smoke. Jackson et al. (2013) reported that exposure to indoor air pollution from the use of biomass fuels for cooking was associated with childhood pneumonia (OR 1.57, 1.06–2.31). Dherani et al. (2008) conducted a meta-analysis using 25 studies which reported that fuel used at home, living environment and behaviour could influence the quality of indoor air in low- and middle-income countries. Dherani et al. (2008) reported that the use of unprocessed solid fuel was associated with childhood pneumonia (OR 1.8, 1.5–2.2) in low- and middle-income countries. Finally, Smith et al. (2000) confirmed a strong association between household biomass fuel and pneumonia episodes in children (OR 3.02, 2.11–4.31) in low- and middle-income countries.

## Discussion

Over the past 17 years, an increasing number of studies have investigated the role of indoor air pollution and its effect on childhood pneumonia in children below the age of 5. Twelve of these studies have mainly focused on PM_2.5_ (Mortimer et al. 2017; Smith et al. 2011; Bassani et al. 2010; Broor et al. 2001; Bruce et al. 2013; Buchner and Rehfuess 2015; Dherani et al. 2008; Dhimal et al. 2010; Dionisio et al. 2012; Howie et al. 2016; Jackson et al. 2013; Karki et al. 2014; Kelly et al. 2015; Mahalanabis et al. 2002; PrayGod et al. 2016; Ram et al. 2014; Rudan et al. 2008; Sharma et al. 1998; Shibata et al. 2014; Smith et al. 2000; Sonego et al. 2015; Zar and Ferkol 2014), solid fuels and general risk factors within low- and middle-income countries. This has led to a greater understanding and better management of the disease over the years. Pneumonia-related deaths are decreasing. In 2000, the mortality rate was 1.7 million but has fallen to 920,000 globally in 2015 (UNICEF 2016). Undeniably, compared to mortality rates in other common paediatric diseases such as measles, HIV and malaria, pneumonia-associated mortality rates have had a significantly slower rate of reduction (UNICEF 2016).

Results from this review confirm the role exposure to indoor air pollution plays in childhood pneumonia. Ninety per cent of all the studies included in this review reported that solid fuel use was significantly associated with an increased incidence of childhood pneumonia. However, 10% reported no significant association between solid fuel use and the risk of childhood pneumonia. These discrepancies in the results might be due to the different study designs and sample sizes used. Also, when indoor air pollution is measured, the association fails to reach a statistical significance. However, when solid fuel is used as a proxy, a larger more significant association is observed. This could be due to equipment used in the measurement of pollution, study power and also lower-quality study designs. Nonetheless, there is evidence suggesting a link between solid fuel use and incidence of pneumonia in children. There is a need to improve the study designs: first, to investigate the solid fuel–meditated pollutant components for each fuel type and then investigate the relationship with pneumonia incidence.

Studies, where CO was used as a proxy for pollution, showed no association on childhood pneumonia. Although solid fuel use has been shown to have a strong association with increased child CO exposure, there is no evidence to suggest the link to disease incidence. However, more studies need to be done to elucidate the validity of CO exposure as a metric for pneumonia incidence in children, especially given the emergence of improved methods of measuring indoor CO exposure (Klasen et al. 2015; Zhang et al. 2013).

Children with higher exposure to particulate matter were at a higher risk of developing pneumonia compared to children with less exposure. It is important to note that this association of PM_2.5_ and incidence of childhood pneumonia were observed when biomass fuel use was used as a proxy for PM_2.5_ and not when PM_2.5_ was directly measured (Karki et al. 2014; Rudan et al. 2008; Sharma et al. 1998; Sonego et al. 2015). PM_2.5_ as an effective exposure metric in health outcome studies has been contested (Patange et al. 2015). Furthermore, results from this review showed that cooking and living spaces had higher levels of PM_2.5_ in case houses compared to controls (Karki et al. 2014; PrayGod et al. 2016; Shibata et al. 2014). Crowding was more common in the houses of cases compared to controls (Ram et al. 2014). All these suggest a link between polluted indoor air and pneumonia incidence. However, these studies could not directly link measured PM_2.5_ with childhood pneumonia after multivariate analysis did not reach a statistical significance. It is, therefore, important to look closely at the components of PM_2.5_ that might be better exposure metrics to investigate. For example, black carbon (BC), which is produced following the incomplete combustion of solid fuels, has shown a higher association with disease incidences in studies where the direct association between PM_2.5_ did not show more association (Geng et al. 2013; Wang et al. 2013). So far, no study has looked at BC and childhood pneumonia. Therefore, studies looking at the influence of indoor air pollution and childhood pneumonia in the future should account for individual components of PM_2.5_ such as PM_1_, where possible, and BC (Baumgartner et al. 2014).

Other risk factors that showed possible association with childhood pneumonia were season where there was an increased risk of pneumonia (OR 4.2, 3.1–5.7) in the rainy season compared to the dry season (Kelly et al. 2015), and bed sharing with someone with a cough which increased the risk of severe pneumonia (OR 5.1, 3.2–8.2) and non-severe pneumonia (OR 7.3, 4.1–13.1) (Howie et al. 2016). Also, undernutrition was associated with childhood pneumonia (OR 8.7, 4.2–17.8) (Howie et al. 2016). Finally, crowding and building materials showed a significant association with childhood pneumonia (Ram et al. 2014).

### Strengths and limitation

This review looked at the individual constituents of indoor air pollution and how they affect pneumonia in children under 5. The search strategy was designed to be extensive and, at all stages, minimise chance and bias by excluding studies with fewer than 100 patients. The review was not restricted by language, study design or year of publication and took into account published and unpublished materials from nine databases. Studies, where secondary data was used, should be interpreted with care because data from national surveys were collected for other objectives. Studies where caregivers or study personnel followed an outcome definition of pneumonia with at least one precise sign of as described by the WHO were included. There was no standard definition of pneumonia, which could mean some of the impacts of indoor air pollution may remain under- or overreported.

A limitation with comparing all of these studies is the lack of standardisation of methods used. The air quality measurements could have been influenced by the equipment and study design used in each study. Not all air quality equipment and study design can give a comprehensive overview of the indoor environment, which will often require continuous measurements with detailed activity monitoring.

Seven of the 12 studies based their diagnosis on a qualified personnel’s objective assessment of pneumonia, with two looking at symptoms. The risk of disease misclassification bias cannot be ruled out given that none of these studies used radiological confirmation of pneumonia. Probably, this could be the reason for some of the findings of the review with regard to association between exposure to pollutants and childhood pneumonia as an outcome. Methodologically, none of these studies was fit to assess causality or dose-effect relationship. Majority of the studies were conducted in rapidly progressing peri-urban areas. Nonetheless, none of them investigated the interaction between outdoor and indoor air quality and their potential combined impacts on childhood pneumonia.

### Research gaps identified

Air pollution research and guidelines have exclusively focused on PM_2.5_ and CO rather than the sources or components including those proposed by the World Health Organization (WHO 2017). Emerging research shows that BC has a higher health-related impact compared to PM_2.5_ (Baumgartner et al. 2014). PM_2.5_ is a complex pollutant consisting of components such as sulphates, nitrates and black carbon (US-EPA 2017). Previous studies have shown that an intervention targeted generally at PM reduction does not necessarily lead to a corresponding decrease in all the components of PM (Aung et al. 2016; Kar et al. 2012; Preble et al. 2014). For example, the development of improved cookstoves aimed at reducing PM mass resulted in higher BC concentrations (Aung et al. 2016; Kar et al. 2012; Preble et al. 2014). Propositions have been made to include BC as an outdoor air quality indicator (Baumgartner et al. 2014). BC, as suggested by other researchers, could be significant in the future as an exposure assessment tool for health studies because currently, we might be underestimating the true health effect of indoor air pollution by focusing largely on PM_2.5_. Also, if BC has a higher health impact than PM_2.5_, its measurement will involve smaller sample sizes and will lead to more accurate health estimates of the impact of indoor air pollution in children under 5. This will impact on policies and interventions.

## Conclusion

Findings from this review indicate that measurements and definitions of exposures and outcomes are unclear and so is the current evidence of indoor air pollution and pneumonia in children. The use of proxy indicators such as solid fuel has suggested an association; however, other proxy indicators such as CO and PM_2.5_ have not shown any associations, and this may be as a result of methods used. We recommend that future studies should have a standardisation of methods to improve the comparability of studies. There were no studies investigating the effect of BC and PM_1_ on childhood pneumonia. Similarly, the interaction between outdoor and indoor air pollution has not been investigated. Therefore, we propose pilot studies to fill these gaps in regions of high incidence of childhood pneumonia. A stepwise approach starting with hospital cohorts and moving to survey data and then birth cohorts will altogether culminate in filling these knowledge gaps. Preferably, factors influencing pneumonia incidence, disease onset and lasting health outcomes should be ascertained in all cases of respiratory infections in children under age 5. Also, future studies should try to understand how season, bed sharing, undernutrition, crowding and building materials can influence the risk of childhood pneumonia. This may help inform more targeted strategies to reducing the burden of pneumonia in this age group. Studies addressing these research gaps will be important to inform strategies aimed at reducing the incidence of childhood pneumonia in low- and middle-income countries.

## Appendix 1. Search terms

### PubMed

#### Indoor air pollution

(((“Air Pollution, Indoor/adverse effects”[Mesh] OR “Air Pollution, Indoor/analysis”[Mesh] OR “Air Pollution, Indoor/statistics and numerical data”[Mesh])) OR “Pollution”[Mesh] “Tobacco Smoke Pollution”[Mesh]) OR “Air Pollution, Indoor”[Mesh]

#### Pneumonia

“Pneumonia”[Mesh] OR “Pneumonia, Ventilator-Associated”[Mesh] OR “Pneumonia, Bacterial”[Mesh] OR “Pneumonia, Viral”[Mesh] OR “Pneumonia, Staphylococcal”[Mesh] OR “Pneumonia, Rickettsial”[Mesh] OR “Pneumonia, Mycoplasma”[Mesh] OR “Pneumonia, Pneumococcal”[Mesh] OR “Pneumonia, Aspiration”[Mesh] OR “Pulmonary Eosinophilia”[Mesh] OR “Idiopathic Interstitial Pneumonias”[Mesh] OR “Idiopathic Pulmonary Fibrosis”[Mesh] OR “Lung Diseases, Interstitial”[Mesh] OR “Bronchopneumonia”[Mesh]

#### Children

(“Child, Preschool”[Mesh]) AND “Child”[Mesh]

((((“Air Pollution, Indoor/adverse effects”[Mesh] OR “Pollution”[Mesh] OR “Air Pollution, Indoor/analysis”[Mesh] OR “Air Pollution, Indoor/statistics and numerical data”[Mesh])) OR “Tobacco Smoke Pollution”[Mesh]) OR “Black carbon”[Mesh] OR “Air Pollution, Indoor”[Mesh] AND “Pneumonia”[Mesh] OR “Pneumonia, Ventilator-Associated”[Mesh] OR “Pneumonia, Bacterial”[Mesh] OR “Pneumonia, Viral”[Mesh] OR “Pneumonia, Staphylococcal”[Mesh] OR “Pneumonia, Rickettsial”[Mesh] OR “Pneumonia, Mycoplasma”[Mesh] OR “Pneumonia, Pneumococcal”[Mesh] OR “Pneumonia, Aspiration”[Mesh] OR “Pulmonary Eosinophilia”[Mesh] OR “Idiopathic Interstitial Pneumonias”[Mesh] OR “Idiopathic Pulmonary Fibrosis”[Mesh] OR “Lung Diseases, Interstitial”[Mesh] OR “Bronchopneumonia”[Mesh] AND (“humans”[MeSH Terms] AND ((“infant”[MeSH Terms] OR “child”[MeSH Terms] OR “adolescent”[MeSH Terms]) OR “children nder 5”[Mesh] OR “infant, newborn”[MeSH Terms] OR “infant”[MeSH Terms] OR “infant”[MeSH Terms:noexp] OR “child, preschool”[MeSH Terms])))

### Embase search

exp Infant/ or infant$.mp. or infancy.mp. or newborn$.mp. or baby$.mp. or babies.mp. or neonat$.mp. or exp Child/ or child$.mp. or kid.mp. or kids.mp. or toddler$.mp. or exp Adolescent/ or adoles$.mp. or teen$.mp. or boy$.mp. or girl$.mp. or exp Pediatrics/ or pediatric$.mp. or paediatric$.mp. or paediatric$.mp. or young people.mp.

And

Pneu$.mp. or exp Pneumonia

And

Air pollution/ or indoor air pollution/ or indoor air quality.mp

### Web of Knowledge

Infant or infant or infancy or newborn or baby or babies or neonat or child or child or kid or kids or toddler or adolescent or adolesc or teen or boy or girl or pediatrics or pediatric or paediatric or paediatric or young people

AND

pneu or pneumonia

AND

Air pollution or indoor air pollution or carbon monoxide or carbon mono or carbon dioxide or carbon diox or nitrogen monoxide or nitrogen dioxide or particulate matter or sulphur dioxide or ozone or volatile organic compounds or black carbon

### CINAHL

“(indoor air quality OR indoor air pollution) AND pneumonia AND (children under 5 OR children under five OR children)”

### Scopus

Pneumonia AND indoor air pollution AND children under 5

## Appendix 2

**Table 4:** Pico table of screening criteria for titles and abstracts

Criterion	Guidance notes	Decision
1. Year	No restriction on date	
2. Study design	To include: cross-sectional studies, case-control studies, longitudinal observation studies (prospective and retrospective cohort and longitudinal studies) and systematic reviews (meta-analysis/reviews/reviews of reviews)	
3. Type of participants	Studies involved children aged 0–5 years old	
4. Health outcomes	The study focused on pneumonia as the primary health outcome	
5. Exposure	Does the study look at indoor air pollution as primary exposure	

## Appendix 3. Newcastle-Ottawa Quality Assessment Scale case-control studies

Note: A study can be awarded a maximum of one star for each numbered item within the Selection and Exposure categories. A maximum of two stars can be given for Comparability.

### Selection

1. Is the case definition adequate?
  - Yes, with independent validation
  - Yes, e.g. record linkage or based on self-reports
  - No description
2. Representativeness of the cases
  - Consecutive or obviously representative series of cases
  - Potential for selection biases or not stated
3. Selection of controls
  - Community controls
  - Hospital controls
  - No description
4. Definition of controls
  - No history of disease (endpoint)
  - No description of source

### Comparability

1. Comparability of cases and controls on the basis of the design or analysis
  - Study controls for _______________ (Select the most important factor.)
  - Study controls for any additional factor (This criteria could be modified to indicate specific control for a second important factor.)

### Exposure

1. Ascertainment of exposure
  - Secure record (e.g. surgical records)
  - Structured interview blinded to case/control status
  - Interview not blinded to case/control status
  - Written self-report or medical record only
  - No description
2. Same method of ascertainment for cases and controls
  - Yes
  - No
3. Non-response rate
  - Same rate for both groups
  - Non-respondents described
  - Rate different and no designation

## Appendix 4. Newcastle-Ottawa Quality Assessment Scale cohort studies

Note: A study can be awarded a maximum of one star for each numbered item within the Selection and Outcome categories. A maximum of two stars can be given for Comparability.

### Selection

1. Representativeness of the exposed cohort
  - Truly representative of the average _______________ (describe) in the community
  - Somewhat representative of the average ______________ in the community
  - Selected group of users, e.g. nurses, volunteers
  - No description of the derivation of the cohort
2. Selection of the non-exposed cohort
  - Drawn from the same community as the exposed cohort
  - Drawn from a different source
  - No description of the derivation of the non-exposed cohort
3. Ascertainment of exposure
  - Secure record (e.g. surgical records)
  - Structured interview
  - Written self-report
  - No description
4. Demonstration that outcome of interest was not present at the start of study
  - Yes
  - No

### Comparability

1. Comparability of cohorts on the basis of the design or analysis
  - Study controls for _____________ (select the most important factor)
  - Study controls for any additional factor (This criteria could be modified to indicate specific control for a second important factor.)

### Outcome

1. Assessment of outcome
  - Independent blind assessment
  - Record linkage
  - Self-report
  - No description
2. Was follow-up long enough for outcomes to occur
  - Yes (select an adequate follow-up period for outcome of interest)
  - No
3. Adequacy of follow-up of cohorts
  - Complete follow-up—all subjects accounted for
  - Subjects lost to follow-up unlikely to introduce bias—small number lost ≥ 70% (select an adequate %) follow-up, or description provided of those lost
  - Follow-up rate < 70% (select an adequate %) and no description of those lost
  - No statement

## Appendix 5

**Table 5:** Risk of bias assessment tool (Newcastle-Ottawa scale)

Domain (source of bias)	Assessment	Risk of bias
Selection (representativeness of the sample)	Adequate case definition with independent validation (A)	Low
	Consecutive or obviously representativeness (B)	Moderate
	Selection of community participants (C)	High
	No description of sampling strategy (D)	Unclear/high
Selection (sample size)	Justified and satisfactory (A)	Low
	Not justified (B)	High
Detection (exposure)	Validated measurement tool (A)	Low
	Tool described but non-validated (B)	High
	Tool not described (C)	Unclear/high
Confounding	Adjusted for confounders (A)	Low
	No adjustment for confounders (B)	High
(Detection) Outcome assessment	Syndromic (A)	–
	Presumptive (B)	–
	Both (C)	–
	No description (D)	–

## Appendix 6

**Table 6:** Risk of bias assessment for each study

No.	Country	Name/year of study	Selection (sampling)	Selection (sample size)	Detection (exposure)	Control confounders	Detection (outcome assessment)
1	India	Broor et al. (2001)	A	A	B	A	A
2	Botswana	Kelly et al. (2015)	A	A	B	A	A
3	India	Sharma et al. (1998)	A	A	A	A	A
4	Nepal	Karki et al. (2014)	A	A	A	A	B
5	India	Mahalanabis et al. (2002)	A	A	A	A	B
6	Bangladesh	Ram et al. (2014)	A	A	A	A	B
7	India	Bassani et al. (2010)	A	A	A	A	B
8	Gambia	Dionisio et al. (2012)	A	A	B	A	B
9	Gambia	Howie et al. (2016)	A	A	A	A	B
10	Tanzania	PrayGod et al. (2016)	A	A	A	A	B
11	Nepal	Dhimal et al. (2010)	A	B	A	A	C
12	Indonesia	Shibata et al. (2014)	A	A	B	A	C
13	Malawi	Mortimer et al. (2017)	B	A	A	A	A
14	Guatemala	Smith et al. (2011)	A	B	A	A	A
